# History of Human Challenge Studies

**DOI:** 10.1007/978-3-030-41480-1_2

**Published:** 2020-08-19

**Authors:** Euzebiusz Jamrozik, Michael J. Selgelid

**Affiliations:** 3 Monash Bioethics Centre, Monash University, Melbourne, VIC Australia; 4 Department of Medicine, Royal Melbourne Hospital, University of Melbourne, Melbourne, VIC Australia; 5 Nuffield Department of Population Health, Wellcome Centre for Ethics and the Humanities and The Ethox Centre, University of Oxford, Oxford, UK; 6 Monash Bioethics Centre, Monash University, Clayton, VIC Australia

## Abstract

The intentional infection of human beings with pathogens with the aim of achieving benefits (chiefly, the prevention of more severe disease) has occurred for centuries; the (semi-)systematic testing and recording of such methods dates to the 18th Century in England.

## Experimental Infection in the 18th–19th Century

The intentional infection of human beings with pathogens with the aim of achieving benefits (chiefly, the prevention of more severe disease) has occurred for centuries; the (semi-)systematic testing and recording of such methods dates to the 18th Century in England (Halsband 1953; Weiss and Esparza 2015). Although the credit for initiating a modern science of vaccination is usually accorded to Edward Jenner (1749–1823), who pioneered the use of cowpox (cow giving rise to the *vache* in vaccine, a term coined by Jenner) to prevent smallpox, variolation (sometimes referred to as ‘inoculation’, i.e., the prevention of smallpox by injection or insufflation of material believed to produce a mild infection and thus convey an attenuated risk of the disease) began much earlier, in Asia and the Eastern Mediterranean, and was introduced to England and North America in the early 18th Century (Timonius and Woodward 1714; Halsband 1953, Gross and Sepkowitz 1998; Weiss and Esparza 2015). Furthermore, that prior infection with cowpox protected humans against infection with smallpox was widely believed in cattle farming communities in England (and elsewhere) long before Jenner’s experiments; at least one English farmer, Benjamin Jesty, is known to have intentionally infected members of his family with cowpox as a means of preventing smallpox in 1774 (i.e., 25 years before Jenner’s experiments) (Gross and Sepkowitz 1998). Yet, although there were some earlier ‘trials’ of smallpox variolation and cowpox vaccination, Jenner’s testing of the cowpox vaccine was more systematic, and involved intentional exposure to smallpox after vaccination (with cowpox) to test efficacy in 1796. However, unlike modern human challenge studies, Jenner’s investigations in the late 18th Century did not involve (i) systematic study of the methods required to induce disease safely and reliably in humans or (ii) the testing of preventive/therapeutic interventions against a reliable model of infection.

One of Jenner’s teachers had been the prominent Scottish surgeon John Hunter, a local pioneer of smallpox variolation (which carried higher risks than the later practice of vaccination) (Turk and Allen 1990). Although Hunter is credited with many positive achievements, he has also become infamous for his attempt to prove his (later falsified) theory that gonorrhoea and syphilis were in fact the same disease. In 1767, Hunter used an experimental (challenge) technique: the injection of “venereal matter” from a patient with gonorrhoea into the penis of a single research subject (Dempster 1978). Though it is sometimes claimed, even in recent times (Gladstein 2005), that Hunter himself was the subject, there is no contemporaneous evidence to support this theory, and it appears more likely that Hunter experimented on another individual—especially since it was known that he had attempted to transmit gonorrhoea to others via inoculation of the skin (Dempster 1978). Importantly, the research subject developed evidence of syphilis, which Hunter took (erroneously) as evidence in favour of his theory (that gonorrhoea and syphilis were the same disease); it now appears more likely that the patient with gonorrhoea from whom the sample was obtained was also infected with syphilis. Thus, the experiment was scientifically flawed and (although mercury-based treatment was provided for the experimental syphilis infection (Wright 1981)) arguably carried significant risks that many would consider unacceptably high—especially on the assumption that this was not a case of self-experimentation (self-experimentation is discussed further below).

During the 19th Century there were significant developments in microbiological understanding of infectious disease. Towards the end of the century in particular, challenge experiments generally became more systematic (discussed below in ‘Early challenge studies with vector-borne diseases’). However, from 1800**–**1880, several experiments that would now be judged highly unethical took place in Europe (Macneill 2010). Irish, German, and Russian physician-investigators injected infectious material from patients with gonorrhoea and syphilis into children and babies, and at least one baby died as a result. Although these investigations did appear to confirm the transmission of such infections, the studies were poorly controlled (due to the rudimentary knowledge of microbiology and lack of available treatments at the time, and perhaps also to the callousness of the investigators). The use of, and harm to, minors (even though some were teenagers who were said to have agreed to participate), furthermore, struck some physicians of the time as immoral and likely unnecessary (Macneill 2010).

One reason it was not necessary to experiment on others (including minors) is that challenge studies can involve self-experimentation, which—especially under such uncontrolled and uncertain conditions—might be considered ethically preferable to the recruitment of others. For example, two scientists deliberately infected themselves with cholera bacteria in 1892. One developed clinical cholera, and this was taken as significant evidence linking the microbe with the disease (Benyajati 1966). Another early challenge study, testing a typhoid vaccine in two ‘Officers of the Indian Medical Service’ took place in 1896. Though few details are supplied, if these individuals were medically trained and aware of the details of the study then they may have been able to provide (what would now be considered) proper informed consent to the risks to which they were exposed (Wright 1896).

## Early Challenge Studies with Vector-Borne Diseases

In the late 19th and early 20th Century additional early challenge experiments began to occur on a larger scale and (generally) with increasing scientific rigor. Challenge studies investigating what would now be referred to as vector-borne diseases (e.g., yellow fever, malaria, and dengue) were particularly prominent at the time, and often conducted in endemic countries (in contrast to later challenge studies which have been predominantly conducted in non-endemic countries). Famous early examples of such experiments include (i) the failed attempts by Carlos Finlay to transmit yellow fever from symptomatic patients to healthy individuals in Cuba from 1881**–**1893^1^ (Finlay 1886, 1937; Clements and Harbach 2017) and (ii) the successful transmission of malaria via infected mosquitoes in Italy in 1898 by Battista Grassi (Grassi et al. 1898; Capanna 2006). The latter study provided the first experimental evidence that malaria was transmitted to humans by mosquitoes.^2^ Since malaria was, at that time, endemic to much of Italy (potentially casting doubt on Grassi’s findings, because individuals exposed to mosquitoes during the trial could have contracted the disease elsewhere), a similar experiment was repeated in London (with infected mosquitoes transported from Italy) by Patrick Manson in 1900 (Manson 1900). Manson infected two volunteers (thought to include his son), and successfully cured the induced infection by administration of quinine (Cox 2010).

Elsewhere during the same period, other early research on yellow fever employed challenge study techniques, though such efforts were sometimes unsuccessful and/or harmful. In 1897, Giuseppe Sanarelli, an Italian physician in Uruguay, claimed to have isolated a bacterial cause of yellow fever (now known to be caused by a virus) and injected a culture of these bacteria into 5 hospital patients, perhaps without their knowledge or consent, of whom 3 died (Lederer 2008). The famous Canadian physician William Osler condemned these experiments in the following terms:

To deliberately inject a poison of known high degree of virulency into a human being, unless you obtain that man’s sanction, is not ridiculous, it is criminal. (Sternberg 1898)

Beyond Osler’s sentiment regarding the importance of obtaining a person’s sanction (i.e., consent) to be challenged, many contemporary readers will additionally object to Sanarelli’s apparent callous disregard for the safety of his ‘research subjects’. While modern HCS standardly involve healthy volunteers, Sanarelli’s subjects were neither healthy (being hospital patients) nor volunteers. The virulence of the organism is a more complex issue: the ‘poison’ in questions was, if not *known* to be highly virulent (since this was being ‘tested’), then at least *expected* to be highly virulent (although in retrospect Sanarelli was clearly ill-informed about the infectious agent he thought he was administering). It is noteworthy, however, that other researchers at the time were also using potentially highly virulent forms of the (presumed) agent of yellow fever, albeit with greater care, less harm, and ultimately greater scientific success.

In the same year (1897), in Mexico, a Dr. Ruis injected the blood of yellow fever patients into 3 individuals (whether they consented is unknown) without producing symptoms of disease. Ruis’ unsuccessful experiments pre-dated the successful and larger scale studies led by Walter Reed (Reed 1902). In 1900 experiments by Reed and other members of the Yellow Fever Commission in Cuba demonstrated the transmission of yellow fever to healthy volunteers (i) by injection of blood from confirmed cases and (ii) by mosquitoes fed on confirmed cases. Reed’s research ultimately led to the development of methods to prevent infection by avoiding mosquitoes (Reed et al. 1900; Lederer 2008; Clements and Harbach 2017).

Reed is frequently credited for establishing a prototype of informed consent for research because subjects in these (what would now be described as) yellow fever challenge studies were asked to sign a contract that outlined the expected risks of the research. The contract also entailed payment of $100 (USD)—the equivalent of more than $3000 in 2019—for two months’ participation in the research, which was doubled ($200, equivalent to ~$6000) for those who contracted yellow fever (Lederer 2008; Clements and Harbach 2017). The contract also made note of the high background risk of contracting yellow fever in Cuba at the time, and the relative benefits of high quality medical care for those infected in the course of the research (versus being infected in an uncontrolled fashion with less medical oversight) (Lederer 2008). Although no research subjects in these initial experiments died, the majority contracted yellow fever (in some cases with severe symptoms). One member of the research team (Jesse Lazear) died from yellow fever despite the best available care (Lederer 2008). It has been suggested that Reed’s relatively scrupulous proto-consent procedures were motivated by his awareness of earlier criticisms of Sanarelli (Lederer 2008). It has nevertheless been argued that Reed’s consent form did not sufficiently emphasise the risk of death due to experimental infection with yellow fever (Chaves-Carballo 2013). Subsequent attempts to develop a yellow fever vaccine in Cuba using a challenge study based on the work of Reed’s team (and using a similar consent form) led to three deaths among research participants, public outcry, and the termination of such experiments in Cuba (Chaves-Carballo 2013).

Elsewhere, from 1902 onwards, researchers in Lebanon, Syria, the Philippines, and Australia conducted early challenge studies with dengue virus (which was, at the time, endemic in parts of all four countries, though it has since been eliminated in Australia), which were followed by later studies (from the 1930s onwards) in the USA and Japan (Cleland et al. 1918; Cleland and Bradley 1919; Simmons et al. 1930; Larsen et al. 2015). Early dengue challenge studies sometimes recruited participants who were military personnel and/or medical researchers (including cases of self-experimentation), although Australian researchers also recruited patients from a local asylum (few details regarding recruitment procedures were published), perhaps because of a reported “unexpected difficulty of obtaining volunteers, even with a considerable monetary inducement” (amount not specified) (Cleland and Bradley 1919).

Similarly, early challenge studies of leishmaniasis were conducted in endemic regions of North Africa, the Eastern Mediterranean, and India.^3^ In 1910 investigators published results demonstrating that the inoculation of the skin of research subjects with parasites presumed to cause cutaneous leishmaniasis caused local eruption of the disease (though few details regarding the participants were published) (Nicolle and Manceux 1910; Row 1912). In 1921, the transmission of cutaneous leishmaniasis by sand flies was demonstrated in a human challenge study involving self-experimentation (Théodoridès 1997). Two decades later, after multiple failed attempts (Killick-Kendrick 2013), researchers in India demonstrated the transmission of visceral leishmaniasis (kala-azar) to 5 out of 5 healthy volunteers by infected sand fly bite (Swaminath et al. 1942). The researchers were particularly alert to potential challenges of conducting such research in endemic areas, including (i) the potential role of prior immunity among participants from endemic regions (as a result of previous exposure) and (ii) the possibility that participants might be bitten by other insects and/or infected with leishmania during the study. As a result, they recruited volunteers from a nearby non-endemic area, transported them to a research facility in an endemic area where the experimental infection took place under (by the standards of the time) strict isolation from contact with other insects, and returned them to a non-endemic area for longer term observation. Volunteers were also ‘generously compensated’ with payment of 400 rupees per month (at a time when the usual wage for unskilled labour was less than 200 rupees per month (Palekar 1957)) and provided with curative treatment (Killick-Kendrick 2013). Of note, these human experiments were considered controversial at the time, and previous requests to use prisoners as research subjects were denied (although this may have been in part because local authorities would not permit a reduction in prison sentences as an inducement for inmates to participate (Killick-Kendrick 2013); such inducements had been used in early smallpox vaccine research in the 18th century during which six British prisoners were freed as a reward for participation (Halsband 1953)). Senior British Army officials also refused to approve the use of human participants (multiple animal studies, including challenge studies, were also being conducted) although it has been suggested that the practice was unofficially tolerated, in part because of the significant expected scientific value of the research (Killick-Kendrick 2013).

## Malariotherapy

The 1927 Nobel Prize in Medicine was awarded to the Austrian psychiatrist Julius Wagner-Jauregg for the discovery of malariotherapy (intentional infection with malaria as treatment) for neurosyphilis,^4^ which became a routine treatment in many psychiatric hospitals, administered either by mosquito challenge or by direct injection of human blood infected with malaria (Chopra et al. 1941; Snounou and Pérignon 2013). The use of this ‘therapeutic’ malaria infection was widespread in Europe, North and South America, and India (Chopra et al. 1941)^5^ until the 1940s when penicillin was discovered as an effective treatment for syphilis (Frith 2012; Snounou and Pérignon 2013). The methods used to ‘prove’ that malariotherapy was effective for neurosyphillis appear quite rudimentary in comparison with 21st Century science (e.g., because the many case series published at the time lacked control subjects), and any attempt to undertake a modern, retrospective review of malariotherapy would inevitably be subject to possible biases, making it difficult to draw firm conclusions (Austin et al. 1992). Some patients died after receiving malariotherapy but, again, it is difficult to know how many of these cases were due to malaria infection itself, as opposed to other factors, including complications of neurosyphilis.^6^ In any case, (neuro)psychiatric patients undergoing malariotherapy were effectively used as research subjects by malariologists in de facto human challenge studies that improved scientific understanding of malaria with regards to (i) confirmation that malaria was caused by several different species of *Plasmodium* parasites, (ii) the natural history of disease, (iii) acquired immunity, (iv) transmission dynamics, and (v) the dormant liver stage of vivax malaria^7^ (Shortt et al. 1948; Snounou and Pérignon 2013). At least one investigation reportedly involved consent from the patient and his spouse for a study including liver biopsies (Shortt et al. 1948). These malaria challenge studies undertaken as part of malariotherapy (with or without what would now be considered valid consent to research participation) are still cited by HCS researchers today, including in some of the endemic-region malaria HCS reviewed in detail below (Shekalaghe et al. 2014; Vallejo et al. 2016). Psychiatric malariotherapy patients were, furthermore, the source of parasites used in other studies, including the Stateville Penitentiary program discussed below (Alving et al. 1948; Miller 2013).

While it was not necessary to use malariotherapy patients to study malaria (since at least one similar study with liver biopsy was done contemporaneously in a particularly altruistic healthy volunteer (Shortt et al. 1949)), researchers may have reasoned that malariotherapy patients were ideal candidates for such studies in light of expectations (based on what was known at the time) that they would directly benefit from infection. In retrospect, however, it is questionable whether (i) all patients with neurosyphilis were able to understand and consent to such research (even in cases where consent was sought), and (ii) the persistent use of malariotherapy in the era of penicillin (as a treatment for neurosyphilis) could have been ethically justified.

## Infamous 20th Century Cases and the Rise of Modern Research Ethics

The genesis of modern research ethics (including the development of relevant codes, declarations, guidelines, principles, etc.) is frequently traced to responses to egregious cases of unethical research in the 20th century (Hope and McMillan 2004; Meltzer and Childress 2008). Several of these infamous cases involved intentional infection of research subjects. For example, some of the atrocities committed in the wartime research programs of Germany and Japan during World War II involved intentional infection with pathogens including anthrax, chlamydia, cholera, dysentery, glanders, hantavirus, malaria, paratyphoid, plague, tetanus, tuberculosis, typhoid, and typhus (Tsuchiya 2008; Weindling 2008; Bambery et al. 2015). These programs collectively involved thousands of victims, many of whom died as a result. Prisoners were violently forced to ‘participate’ (with no option to refuse nor effort to seek consent); participation often involved uncontrolled infection with pathogens known to cause severe disease and sometimes involved the torture and murder of those infected (e.g., by vivisection) (Tsuchiya 2008; Weindling 2008). Despite claims that such research aimed to improve measures to protect military personnel from infectious diseases, much of the ‘research’ and/or the procedures involved therein did not have a sound scientific rationale and thus would not have been able to inform the development of such measures, even if it had been conducted in a less violent manner (Tsuchiya 2008; Weindling 2008).

In the USA, contemporaneous war-related research also included recruitment of prisoners for infection challenge studies. Although these were conducted under more humane conditions, the voluntariness of consent, and the legitimacy of recruiting prisoners for research more generally, has since been called into question (Bonham and Moreno 2008; Miller 2013). Of particular relevance to current malaria challenge research, American military research during (and after) WWII included the Stateville Penitentiary experiments (discussed in more detail below), which involved infection of prisoners with malaria (including, in later studies, resistant strains of malaria) (Arnold et al. 1961; Miller 2013).

Later (in 1946**–**48), studies of sexually transmitted infections performed by American researchers in Guatemala involved intentional infection of vulnerable groups (e.g., sex workers, prisoners, soldiers, mentally disabled and institutionalised patients) with pathogens (e.g., bacteria causing syphilis, gonorrhoea, and chancroid) without their knowledge or consent, and also involved deliberately withholding treatment for these infections (Frieden and Collins 2010; Gutmann and Wagner 2012). In the United States, the Willowbrook School study of infectious hepatitis (1950s to 1970s) involved the intentional infection of mentally disabled, institutionalised children with viral hepatitis (which was, at that time, endemic at the school with very high rates of background infection in both ‘patients’ and staff), with the aim of better describing its natural history, and testing preventive and/or therapeutic interventions (Ward et al. 1958; Rothman 1982; Robinson and Unruh 2008).

Although these studies were eventually met with widespread condemnation, there is a consensus in the (limited) academic research ethics literature (discussed below in more detail) that it was not intentional infection per se that made these studies unethical, but rather other issues, particularly those related to (i) lack of or inadequate informed consent, and/or (ii) exploitation and/or brutal treatment of vulnerable populations (Miller and Grady 2001; Hope and McMillan 2004; Miller and Rosenstein 2008; Bambery et al. 2015).

Nevertheless, in high-income countries, studies did continue among populations sometimes described as ‘vulnerable’, e.g., prisoners (Glew et al. 1974) and military personnel (among whom, similarly, it may be more difficult to assure truly voluntary informed consent) (Bonham and Moreno 2008). In a retrospective analysis of the Stateville penitentiary malaria challenge studies conducted by the US military, Franklin Miller contrasts this research program with the (other) abusive wartime research discussed above, noting that (although imprisoned) subjects were invited (not forced) to volunteer, carefully screened for health conditions, and monitored closely during the studies—meaning that such research practices would be largely in accordance with many (subsequently developed) codes of research ethics (Miller 2013). Miller does note, however, that during the research severe adverse reactions and one death occurred (the latter reportedly due neither to infection challenge nor to the antimalarial drugs being trialled), raising plausible but unverifiable concerns that researchers may have been more willing to expose prisoners to higher risks because they were incarcerated (and/or because of the perceived urgency of army research that could save the lives of deployed soldiers) (Miller 2013).

By the 1970s, a consensus was building (although it has perhaps never become unanimous) among research ethicists in developed countries that research among such ‘captive’ groups could be ethically problematic, ultimately resulting in more careful review of research involving military personnel (although this has not necessarily resolved the underlying ethical tensions) (Bonham and Moreno 2008; Miller 2013), and strict regulations regarding research in prisons that eventually curtailed the recruitment of prisoners (Mishkin 2000; Rosenbaum and Sepkowitz 2002).^8^ Some have noted that adult students as a group may sometimes share characteristics with other ‘captive’ groups that might lead to concerns about their ability to consent (although perhaps to a lesser degree)—especially where they are financially or professionally dependent on their academic superiors and/or required to enroll as research participants as part of their studies (Bonham and Moreno 2008). Such considerations may be important for more recent challenge studies, which frequently recruit from student populations.

## Late 20th Century

Later, some post-WWII challenge studies, such as those in the UK Common Cold Unit, involved volunteers from the general population. Although they predated modern ethics regulations, these studies reportedly involved a careful explanation of risks, voluntary consent, and isolation to prevent third party transmission; they did, however, involve risks that were not well characterised at the time, such as the potential for transmission of other pathogens (e.g., in bodily secretions used to administer the infection challenge) for which there were no testing methods available (Tyrrell 1992).

Elsewhere, at least one early (post-WWII) malaria challenge study took place took place in East Africa (an endemic-region), investigating the degree to which sickle cell trait (a genetic condition affecting red blood cells) protects against malaria. In the 1954 publication of this study (Allison 1954), the 30 research subjects are described as adult male volunteers from the Luo people, and it is mentioned that risks were controlled by giving infected subjects “a prolonged course of antimalarial chemotherapy”. Few other details apart from the infection rate in sickle cell trait versus non-sickle trait participants are noted—e.g., the publication records neither the presence nor severity of symptoms among participants, nor any consent process. In 1956 there was also a case of self-experimentation by a single investigator in Nigeria who infected himself with Zika virus and attempted to transmit the virus from himself to laboratory mice (Bearcroft 1956).

While it is possible (perhaps likely) that other endemic region/low-resource country challenge studies were conducted between World War II and 1992 (the date of the first case study reviewed later in this report), our review found that there was a very sparse literature regarding endemic region challenge research during this period, especially as compared to the significant and relatively numerous studies published from the late 19th Century to World War II. This may in part be due to the significant social changes that occurred in endemic regions (many of which were previously controlled by European imperial powers) at the end of the colonial period.

Ethical concerns (and reactions to the egregious cases discussed above) have perhaps contributed to a reluctance to undertake more HCS in LMICs (in addition to any technical difficulties regarding the availability of necessary laboratory infrastructure etc.) because (i) impoverished individuals and communities may be (perceived to be) particularly vulnerable (e.g., to various kinds of harm, exploitation, inducement by monetary payment, etc.), and (ii) valid informed consent may be (perceived to be) more difficult to assure in some populations within LMICs (e.g., because of language barriers, limited educational background, etc.).

In any case, HCS research has been largely concentrated in HICs, even where such research addresses pathogens that are primarily endemic in LMICs. For example, in the latter half of the 20th Century, North American and European researchers developed malaria challenge models, ultimately leading to several parallel research programs (Spring et al. 2014; Friedman-Klabanoff et al. 2019). At the outset, such studies were subject to few regulatory requirements and/or ethical oversight mechanisms. Parasites were obtained from infected human ‘donors’; challenges involved multiple ‘wild-type’ malaria pathogens (rather than, in the case of falciparum malaria, the few well-characterised laboratory strains in widespread use today); and prisoners and/or army personnel featured prominently among early recruitment of participants (see discussion of these groups above) (Friedman-Klabanoff et al. 2019).

Since the 1980s, improvements in scientific techniques as well as rigorous regulatory and ethical oversight have supported the development of multiple HCS research programs, studying a wide range of pathogens predominantly in HICs (Miller 2013; Darton et al. 2015). Studies collectively enrolling tens of thousands of healthy volunteers (Darton et al. 2015; Evers et al. 2015) have been safely conducted with no deaths and very few serious or lasting harms reported among HCS participants (Roestenberg et al. 2012; Darton et al. 2015). Pathogens/diseases studied in such trials have included adenovirus, BCG (bacille Calmette–Guérin—an attenuated form of *M. bovis* used as a tuberculosis vaccine), campylobacter, *Candida albicans*, cholera, coronaviruses, cryptosporidium, *Cyclospora cayetanensis*, cytomegalovirus, dengue, *E. coli*, giardia, hepatitis A & B, hookworm (*Ancyclostoma caninum* and *Necator americanus*), influenza, gonorrhoea, *H. ducreyi*, *H. pylori*, listeria, malaria, norovirus, parainfluenza, parvovirus, pneumococcus, Q fever, respiratory syncytial virus, rhinovirus, rotavirus, scabies, streptococci (non-pneumococcal), *Shigella spp.*, *Strongyloides spp.*, and typhoid. Overall, not only have the vast majority of HCS been conducted in HICs, but most studies have hitherto focused on pathogens that cause disease in HICs (though they may also affect LMICs), rather than those that are primarily endemic to LMICs; for example, rhinovirus (a cause of the common cold) has been the pathogen associated with by far the greatest number of HCS (at least 55 studies enrolling, collectively, >18,000 participants, in both respects more than for any other pathogen) (Kalil et al. 2012; Darton et al. 2015; Evers et al. 2015). HCS have resulted in unique insights into host-pathogen interactions, as well as the accelerated development of beneficial interventions, including for pathogens primarily endemic in LMICs. For example, HCS have played a role in the development of recently approved and/or licensed vaccines against typhoid (Jin et al. 2017), cholera (Tacket et al. 1999), and malaria (Ballou 2009).

## Capacity Building in Low- and Middle-Income Countries

More recently, there have been calls for more HCS in endemic settings (particularly for pathogens that are primarily endemic in LMICs) in order to accelerate vaccine development and test new interventions in the populations at highest risk of relevant diseases (Gibani et al. 2015; Gordon et al. 2017; Baay et al. 2018). Our review identified no HCS conducted in LMICs from 1956 until 1992 when what appears to be the first LMIC HCS in nearly 40 years took place in Thailand (Suntharasamai et al. 1992). Researchers from LMICs have, however, participated in international HCS projects (where the challenge infection takes place in a HIC). For example, Thai researchers furnished mosquitoes infected with malaria parasites for HCS conducted in the USA (Malaria Vaccine Initiative 2016). However, it has been reported that Thai institutions were initially reluctant to conduct vivax malaria HCS in Thailand (partly because Thai authorities were awaiting further evidence of the safety and utility of the challenge model in question) (Malaria Vaccine Initiative 2016).

From 1992 onwards, Thai researchers successfully conducted challenge studies with cholera and *Shigella* in populations of Thai volunteers. Elsewhere, in the last two decades, well-established research centres in Colombia, Tanzania, Kenya, and Gabon have successfully conducted malaria HCS, often in collaboration with HIC HCS researchers (discussed in detail in Chap. 10.1007/978-3-030-41480-1_5). Researchers in more LMICs—including Equatorial Guinea, India, Indonesia, Malawi, Mali, Uganda, and Vietnam—are understood to be considering and/or conducting HCS at present.

Later, we discuss the ethical and scientific case for conducting (more) appropriately designed HCS in endemic LMICs in greater detail (Section “10.1007/978-3-030-41480-1_3”). However, even if there is an especially strong case for conducting such studies, certain (other) ethical issues related to their design and conduct warrant particularly careful attention. This is because (i) HCS may sometimes involve, or at least be perceived to involve, particularly high levels of risks (for participants and third parties) and other burdens for participants (and such studies must therefore be carefully designed and conducted to ensure that expected benefits outweigh risks and burdens), and (ii) local and/or international community acceptance of HCS being conducted in endemic LMICs may be contingent on such studies being designed and conducted to especially high ethical (and scientific) standards, and (iii) certain ethical considerations, though familiar in research ethics discourse, may have particular (underexplored) implications in the context of endemic LMIC HCS. The evaluation of these latter implications may both improve the design and conduct of LMIC HCS and/or provide novel case studies relevant to ongoing debates in research ethics. The remainder of this report summarises ethical and regulatory issues relevant to such studies, including insights from stakeholders interviewed for the current project, followed by a comparative review of LMIC HCS published in 1992**–**2018.

## Footnotes

1 Although Finlay’s overall hypothesis was correct, these experiments failed to demonstrate transmission because the interval between biting infected patients and biting healthy individuals (now known as the ‘extrinsic incubation period’) was too short.

2 Ronald Ross, the English contemporary of Grassi who was awarded the 1902 Nobel prize for identifying mosquito transmission of malaria, had (in 1897) shown that parasites were transmitted from human malaria patients to mosquitoes but used challenge studies in birds (with avian malaria), rather than human challenge, to show the transmission *from* mosquitoes.

3 We are grateful to Dr. Kate Emary for pointing us in the direction of early leishmania challenge studies.

4 The debilitating end-stage of syphilis that was relatively common at the time and had no effective treatment.

5 Interestingly (in the context of this review of endemic-region HCS research) while most malariotherapy programs in Europe and North America reportedly used *P. vivax*, at least one Indian centre used *P. falciparum* (which usually causes a more severe form of malaria) because it was believed that the local population had significant immunity to *P. vivax* that would attenuate the benefits of malariotherapy (see Chopra et al. 1941).

6 However, when falciparum malaria (a more severe form) was used by mistake in malaria-naïve patients for malariotherapy instead of vivax malaria (the milder form of malaria usually used), the mortality rate was much higher (Austin et al. 1992). In contrast, see the use of falciparum in India in the footnote above.

7 First identified in 1948 when a malariotherapy patient reportedly consented to a liver biopsy (see Shortt et al. 1948)—i.e., in a proto-challenge study.

8 In one countervailing consideration, Rosenbaum and Sepkowitz (2002) cite a case of a group of prisoners at (US) Jackson State Prison filing an (unsuccessful) lawsuit arguing that prisoners should have more freedom to participate in research, though this may have been partly because of a view that participation in research would entail thorough medical examination and care, which can be difficult for prisoners to access under usual circumstances.
